# Effect of Guanxin V in animal model of acute myocardial infarction

**DOI:** 10.1186/s12906-021-03211-7

**Published:** 2021-02-22

**Authors:** Xiaoxiao Zhang, Changle Shao, Songyi Cheng, Yao Zhu, Bo Liang, Ning Gu

**Affiliations:** 1 Nanjing University of Chinese Medicine, Nanjing, China; 2 Department of Cardiology, Nanjing Hospital of Chinese Medicine, Affiliated to Nanjing University of Chinese Medicine, Daming Road 157#, Nanjing, 210000 Jiangsu People’s Republic of China; 3 Xuzhou City Hospital of TCM, Xuzhou, China

**Keywords:** Acute myocardial infarction, Ventricular remodeling, Chymase, Mast cells, Syrian hamsters, Traditional Chinese medicine

## Abstract

**Background:**

Acute myocardial infarction (AMI) is the most serious and lethal manifestation of coronary heart disease worldwide, presenting extremely high disability and mortality. Our previous studies have shown that Guanxin V (GXV) could significantly improve the cardiac function and the blood flow dynamics, and reduce serum levels of inflammatory factors in AMI rats, thus triggering ventricular remodeling (VR) at post-AMI.

**Methods:**

An in vivo AMI model was established in Syrian hamsters by performing the ligation of the left anterior descending coronary artery. Syrian hamsters were randomly divided into four groups, namely Sham operation group (*n* = 12), AMI group (*n* = 12), GXV group (GXV 6 g/Kg/d, *n* = 12), and Tranilast group (Tra 105 mg/Kg/d, *n* = 12). Drug intervention was conducted for consecutive 8 weeks. Relative biological indicators were measured in the 4th and 8th week, respectively.

**Results:**

Cardiac functions were improved, and the infarcted size and heart weight index were limited in Syrian hamsters of GXV and Tra groups compared with those in AMI group. Furthermore, GXV was able to decrease the number of mast cells and chymase level in Syrian hamsters with AMI. Administration of GXV remarkably inactivated the renin-angiotension-aldosterone system, and alleviated myocardial fibrosis and cardiomyocyte apoptosis, thus slowing down VR at post-AMI.

**Conclusion:**

GXV slows down the process of VR at post-AMI by reducing chymase level and mast cells number, as well as inactivating the reninangiotension-aldosterone system..

**Supplementary Information:**

The online version contains supplementary material available at 10.1186/s12906-021-03211-7.

## Background

Acute myocardial infarction (AMI) is a severe and lethal coronary heart disease with extremely high disability and mortality [1]. Ventricular remodeling (VR) is first proposed by Tennant and Wiggers, which involves pathological changes of ventricular dilatation, cicatrization and structural changes of the left ventricle [2]. AMI triggers morphological changes of myocardium and myocardial fibroblasts due to oxidative stress, inflammatory response, neuroendocrine regulation and other secondary lesions, thus affecting ventricular size, structure and function. Eventually, AMI-induced pathological changes lead to the incidence of VR. The occurrence of VR may further increase the risks of heart failure and malignant arrhythmias, and even cardiogenic death. Therefore, prevention and reversal of VR are beneficial to AMI treatment [3]. Early revascularization, pharmacotherapy, and device-based therapies are currently preferred to AMI patients [4].

Guanxin V (GXV) is produced by the Nanjing Hospital of Chinese Medicine Affiliated to Nanjing University of Chinese Medicine (Nanjing, China). It is applied for the treatment of coronary heart disease, myocardial infarction, and arrhythmia. Main components of GXV are *Codonopsis* (Dang Shen), *Monkey Grass* (Mai Men Dong), *Schizandra Berry* (Wu Wei Zi), *Salvia Miltiorrhiza* (Dan Shen), *Red Peonies* (Chi Shao Yao) and *Rehmannia Root* (Shu Di Huang) (Table 1) [5]. It is reported that *Codonopsis* can inhibit the apoptosis signaling AngII+Leu27-IGFII in cardiomyocytes [6]. *Rehmannia Root* extract can activate the SDF-1α/CXCR4 cascade following AMI, thus triggering angiogenesis by reinforcing the mobilization and migration of endothelial progenitor cells [7]. Steroidal saponins is the main ingredient of *Monkey Grass*, which exerts various pharmacological activities, including cardiovascular protection, anti-inflammation, anti-cancer ability, anti-oxidation and immunomodulation [8]. Another study indicated that Tanshinone IIA inhibits myocardial apoptosis by inducing the expression of miR-133 and restraining the caspase-9 cascade [9]. Crude terpene glycoside components in *Red Peonies* is capable of protecting myocardial ischemia by stimulating cardiac energy metabolism and inhibiting myocardial apoptosis via activating the PI3K/AKT/mTOR signaling pathway [10]. *Schizandra Berry* and its main components protect against cardiovascular diseases mainly through suppressing oxidative stress, cardiomyocyte apoptosis and inflammatory response [11]. Therefore, it is believed that GXV, a traditional Chinese medicine can be applied in the treatment of heart diseases.

**Table 1:** Introduction to the composition of GXV

Common name of the plant	Scientific name of the plant	Latin name of the plant	Plant part	Quantity(g)
Dang Shen	*Codonopsis*	*Codonopsis pilosula* *(Franch.) Nannf.*	Root	200
Mondo Grass, Mai Men Dong	*Monkey Grass*	*Ophiopogon japonicus* *(Linn. f.) Ker-Gawl.*	Tuberous root	100
Wu Wei Zi	*Schizandra Berry*	*Schisandra Chinensis (Turcz.) Baill*	Fruit	50
Dan Shen	*Salvia Miltiorrhiza*	*Salviae Miltiorrhizae Radix et Rhizoma*	Root and rhizome	200
Chi Shao Yao	*Red Peonies*	*Paeoniae Radix Rubra*	Root	200
Shu Di Huang	*Rehmannia Root*	*Rehmannia glutinosa (Gaert.) Libosch. ex Fisch. et Mey*	Tuberous root	200

Tranilast (N-[3′,4′-dimethoxycinnamonyl] anthranilic acid) is an anti-allergic drug that inhibits the release of chemical indicators from mast cells (MC) and basophils [12]. Besides, Tranilast is also reported to prevent against inflammatory response, cell multiplication and ventricular fibrosis [13–15]. It is reported that Tranilast prevents atrial remodeling in a canine model by inhibiting overexpression of TGF-β1 and Rac1 [16]. In a rat model of myocardial infarction, Tranilast inhibits fibrosis and collagen production in cardiac fibroblasts, thus alleviating LV remodeling [17].

Our previous studies have shown that administration of conventional drugs combined with GXV in AMI patients significantly maintains left ventricular structure and improves function compared with those solely treated with the conventional drugs [18, 19]. Animal experimental studies also revealed that multiple preparations containing GXV obviously improve the cardiac structure and function, and suppress inflammatory response in AMI rats [20, 21]. In AMI rats, treatment of GXV combined with valsartan prolongs the survival, which slows down the increase rate of ventricular weight, alleviates pathological changes of myocardial tissues and ventricular structure, and angiotensin II type 1 receptor (AT1R) and upregulates angiotensin II type 2 receptor (AT2R) in the renin-angiotension-aldosterone system (RASS) [22]. A retrospective study demonstrated that Guanxin V is potentially safe and effective for patients with coronary artery disease, which enhances ejection fraction and output, and reveres VR [23]. The above evidences have proven that GXV is a potential natural drug used in adjusting VR at post-AMI. However, the mechanism of GXV on regulating the RAAS remains largely unknown. This study intends to observe the effect of GXV on VR and RAAS at post-AMI and the underlying mechanism.

## Methods

### The preparation of GXV

Main components of GXV are shown in Table 1. All herbs were recorded in the *Pharmacopoeia of the People’s Republic of China* compiled by China Pharmacopoeia Committee. These herbs were purchased from Department of Pharmacy, Nanjing Hospital of Chinese Medicine Affiliated to Nanjing University of Chinese Medicine (Nanjing, China). The authentication of plant materials was identified morphologically by Dr. Li Wen, the chief physician of Nanjing Chinese Medicine Affiliated to Nanjing University of Chinese Medicine. The voucher specimen of this material has been deposited in Department of Cardiology, Nanjing Hospital of Chinese Medicine Affiliated to Nanjing University of Chinese Medicine. *Salvia Miltiorrhiza* and *Schizandra Berry* were extracted by refluxing 4 times the volume of 75% ethanol twice, with 2 h each time. Subsequently, they were mixed with the remaining drugs, and decocted in 10 times the volume of water for 3 times, with 2 h each time, which was finally concentrated to 1000 mL. The preparation procedures were entrusted to the Department of Pharmacy, Nanjing Hospital of Chinese Medicine Affiliated to Nanjing University of Chinese Medicine.

### Reagents

The enzyme-linked immunosorbent assay (ELISA) kits for renin, angiotensin converting enzyme (ACE), chymase, angiotensin I (Ang I) and angiotensin II (Ang II) were obtained from Meimian (Wuhan, China). The primary antibodies against ACE, Chymase and angiotensin II type1 receptor (AT1R) were purchased from Abcam (Cambridge, UK), while those against Collagen I and Collagen III were provided by Bioss (Wuhan, China). Triphenyl tetrazolium chloride (TTC) staining kit was obtained from Sigma (Aldrich, St. Louis, USA). H&E and Masson staining kits were provided by Solarbio (Beijing, China). In situ apoptosis detection kit was obtained from Roche (New Jersey, USA). Goat anti-rabbit antibodies and DAB detection kit were purchased from ZSGB-BIO (Beijing, China). Toluidine blue (TB) staining kit was brought from LEAGENE (Huaibei, China). Acetonitrile and formic acid for ultra-performance liquid chromatography (UPLC) analysis were provided by Sigma. Tranilast capsules were brought from Pharmaceutical Company of China Pharmaceutical University (Nanjing, China).

### UPLC

UPLC was performed to analyze the main ingredients of GXV and medicated sera using the UPLC-Q-TOF/HRMS^E^ system (Waters, Milford, MA, USA). An ACQUITY UPLC BEH C18 (2.1 mm × 100 mm,1.7 μm Waters, Milford, USA) column was used with a mobile phase including A (0.1% formic acid, *v/v*) and B (0.1% formic acid acetonitrile). The gradient elution was applied as follows: 98–40% A and 2–60% B for 0–14 min; 40–2% A and 60–98% B for 14–16.5 min; 2% A and 98% B for 16.5–18 min; 98% A and 2% B for 18–20 min (flow rate: 0.3 ml/min, column temperature: 40 °C).

The high-resolution ion mobility liquid mass spectrometer SNAPT G2-Si Q-TOF/HRMS was used for mass spectrometry analysis in the mode of positive and negative ions using the ESI source. The high-purity N_2_ was utilized as the auxiliary spray ionization and dissolvent gas. The following parameters were applied: Flow rate of drying gas: 10 mL∙min^− 1^; N_2_ temperature: 120 °C; Atomization gas pressure: 310 kPa; Flow rate of dissolvent gas: 900 L∙h^− 1^; Flow rate of cone blowback: 50 L∙h^− 1^; Capillary ionization voltage: 500 V; Cone voltage: 40 V; Collision energy: 40–65 eV and scanning range of quadrupole: 50–1200 Da.

### Experimental animals

Animal procedures were approved by the Animal Ethics Committee, Nanjing University of Chinese Medicine (Nanjing, China). Sixty male Syrian hamsters (110 ± 20 g, 8 weeks old) were provided the Vital River Laboratory Animal Technology Co. LTD (Beijing, China). They were raised in the Laboratory Animal Centre, Nanjing University of Chinese Medicine (12 h light/dark cycle, temperature: 23 ± 1 °C, humidity: 55–60%). Syrian hamsters were given to free accesses to the food and water.

An in vivo AMI model was established in Syrian hamsters by performing the ligation of the left anterior descending coronary artery (LAD) [24, 25]. The hamsters were anesthetized by subcutaneous injection of 2% pentobarbital sodium (4 ml/kg, Sigma). The electrocardiography (Lead ii, ECG-9620P, Shanghai optoelectronic medical instrument Co., LTD, Shanghai, China) was performed by connecting electrodes to subcutaneous extremities of hamsters. After shaving, iodophor disinfection and cutting off the neck skin, hamster trachea was exposed for the following blunt dissection of muscles. Mechanical ventilation was conducted by endotracheal intubation though a ventilator (ALC-V8S, Shanghai Alcott biotechnology Co., LTD, Shanghai, China). Ventilation indexes were set as follows: Tidal volume: 4-5 ml; The ratio of inhalation to respiration 1:2; Respiration rate: 75 times /min. The heart was exposed by cutting off the skin alongside the 3rd to 4th intercostal level of the left chest. Subcutaneous tissues and muscles were bluntly separated layer by layer. After exposure of the heart, LAD was performed by suturing at 2 mm below the lower margin of the left auricle using the 7–0 silk suture. Decreased myocardial motion, pale myocardium and 0.2 mV elevation of ST segment indicated the successful establishment of AMI model in Syrian hamsters. Finally, the exposed chest was sutured layer by layer and the residual gas was squeezed out. Extubation was performed until the heart rhythm was stable. Syrian hamsters in the Sham operation group received the same procedures except for ligation. After animal procedures, 4 U penicillin (Zhongnuo pharmaceutical Co. LTD, Shijiazhuang, China) was subcutaneously administrated for consecutive 3 days to prevent postoperative infection.

### Experimental design

A total of 48/60 (80%) hamsters survived after procedures. According to the postoperative survival, the hamsters were randomly divided into four groups, namely Sham operation group (*n* = 12), AMI group (*n* = 12), GXV group (GXV 6 g/Kg/d, *n* = 12) and Tranilast group (Tra 105 mg/Kg/d, *n* = 12). Hamsters in AMI group, GXV group and Tra group were operated by LAD to establish the AMI. The equivalent doses of GXV and Tranilast administrated in hamsters were converted according to the adult doses of a standard weight. The same volume of normal saline was administrated in those of Sham operation group and AMI group. Intragastric administration was given on the 3rd day postoperatively and once a day for 8 weeks, which was independently performed by the investigator Xiao-Xiao Zhang based on the randomization table who was the only one being aware of the treatment group allocation. Cardiac markers were measured in the 4th and 8th week, respectively. The hamsters were anesthetized by subcutaneous injection of 2% pentobarbital sodium (4 ml/kg) for collecting blood collection from abdominal aorta, and their hearts. All hamsters were sacrificed by cervical dislocation and their carcasses were concentrated destructed by the Laboratory Animal Centre, Nanjing University of Chinese Medicine.

### Determination of cardiac functions

The hamster was anesthetized and fixed at the supine position. M-mode echocardiograph was obtained using a small-animal ultrasound probe (VisualSonics Vevo2100, Toronto, Canada) on the long axis of the parastolic left ventricle. The following indicators were recorded: Left ventricular end-diastolic inner diameter (LVIDd), left ventricular end-systolic inner diameter (LVIDs), left ventricular end diastolic volume (LVEDV), left ventricular end systolic volume (LVESV), ejection fraction (EF), fractional shortening (FS), left ventricular posterior wall end-diastolic thickness (LVPWd) and left ventricular posterior wall end-systolic thickness (LVPWs).

### Detection of heart weight index and myocardial infarction size

The body weight of each hamster was recorded before sacrifice. After humane sacrifice, the heart was harvested and weighed. The heart weight index (HWI) was then calculated: HWI = heart weight / body weight (HW/BW, mg/g). Then, the heart was frozen in − 20 °C for 10 min, sliced into 2 mm sections and stained in 2% tetrazolium chloride at 37 °C. Thirty minutes later, sections were fixed in 10% neutral formalin. Infarcted myocardium turned pale and the remaining normal one was dark red. Stained heart sections were captured and infarcted size was calculated using Image J software (NIH, Bethesda, USA).

### Histopathology and immunohistochemistry

The heart was cut off from its surrounding vessels. The left ventricle of the heart, including infarcted and non-infarcted areas, was taken and fixed in 4% paraformaldehyde for 48 h and paraffin embedded. Sections were sliced into 3-μm thickness.

Histopathological changes and cardiac fibrosis were observed and captured by a microscope (BX51T-PHD-J11, Olympus, Tokyo, Japan) following H&E, and Masson staining.

MCs were stained by TB and counted by the microscope. Paraffin-embedded sections were dewaxed, hydrated, stained with TB working solution for 10–20 min and washed with distilled water for 3 times. Then, the slices were differentiated with 0.5% glacial acetic acid and washed twice with distilled water. At last, slices were dehydrated with 95% ethanol for 3–5 s, dehydrated with anhydrous ethanol for 1 min × 2 times, penetrated with xylene for 2 min × 2 times, and sealed with neutral gum. MCs were stained blue purple, and the nuclei were light blue.

Immunohistochemical staining was performed by the SP method. Paraffin-embedded sections were dewaxed, hydrated and subjected to antigen retrieval in citric acid buffer for 20 min. Non-specific antigens were inactivated by incubation in 3% hydrogen peroxide for 10 min. After blockage in normal goat serum for 20 min, slices were incubated with corresponding primary antibodies at 4 °C overnight. The biotinylated secondary antibody (IgG) was applied as the negative control. On the next day, slices were washed in phosphate buffer saline (PBS) for 3 times, incubated with horseradish peroxidase-labeled streptomycin working solution (S-A/HRP) at 37 °C for 20 min, and washed in PBS for 3 times. The mixture of color reagent A, B, C (a drop of each, respectively) and 1 ml of distilled water were prepared and applied on the sections. Sections were then thoroughly washed for 6 min, sealed by neutral resin and finally captured using a microscope.

### TUNEL/DAB immunofluorescence

Paraffin-embedded left ventricle heart sections (including infarcted and non-infarcted areas) were dewaxed, dehydrated and incubated with protease K (20 μg/ml dissolved in Tris/HCl, pH 7.4 ~ 8.0) at room temperature for 15–30 min. After washing in PBS twice, sections were reacted with 50 μl of TUNEL reaction mixed solution in a wet box at 37 °C for 1 h. Glass slides were covered to prevent evaporation and ensure the evenly distribution of TUNEL reaction mixture. TUNEL-positive staining was then analyzed under a fluorescence microscope after 3 times washing with PBS. Subsequently, sections were incubated with 50 μl of conversion agent-POD in the wet box at 37 °C for 30 min. Incubation of 50 ~ 100 μl of DAB substrate solution at room temperature for 10 min was conducted following 3 times washing in PBS. At last, nuclei were counterstained by hematoxylin, sealed and captured.

### Western blotting (WB) analysis

Total protein was extracted from the left ventricle heart tissues (including infarcted and non-infarcted areas) of Syrian hamsters. Protein concentration was determined by the bicinchoninic acid (BCA) protein assay kit (Beyotime, Nanjing, China). The concentration of each sample was adjusted to the equal one by adding the calculated loading buffer. Separated proteins were transferred onto polyvinylidene fluoride membranes (PVDF, Millipore, Billerica, USA) after the 10% SDS-polyacrylamide gel electrophoresis. Non-specific antigens were blocked using the 5% bovine serum albumin (BSA, Beyotime, Nanjing, China) for 1 h at RT. Afterwards, membranes were reacted with the diluted primary antibodies (1:1000) at 4 °C overnight, washed with TBST, and reacted with the diluted horseradish peroxidase-conjugated secondary antibodies for 1 h at RT. Protein bands were visualized by the enhanced chemiluminescence (ECL) detection regent (Millipore, Bedford, USA) and a chemiluminescence image analyzer (GE Healthcare, Uppsala, Sweden). Image J software was used to analyze the bands.

### ELISA

Relative levels of renin, ACE, chymase, Ang I and Ang II in serum and the left ventricle heart tissues (including infarcted and non-infarcted areas) of Syrian hamsters were detected by ELISA according to the manufacturer’s recommendations.

### Statistical analyses

All values were expressed as mean ± standard deviation. GraphPad Prism 8.0 (GraphPad Software Inc., San Diego, CA, USA) software was used for statistical analyses. Two-way ANOVA was used to compare the differences between groups. *P* < 0.05 was considered as statistically significant.

## Results

### UPLC profile and constituent contents of GXV

The main components of medicated sera of GXV were analyzed by comparing with the second-order chromatogram and serum pharmacochemistry method. The total ions chromatograph (TIC) was a chromatogram generated after collecting all ions, which had a high universality. Medicated sera of GXV mainly contained 15 components, including Codonopiloside A, Lobetyolin, dihydrocatalpol, Ctlpol, RehmanniosideA, and others (Table S1).

### GXV effectively improved cardiac function in AMI hamsters

**Fig. 1:**
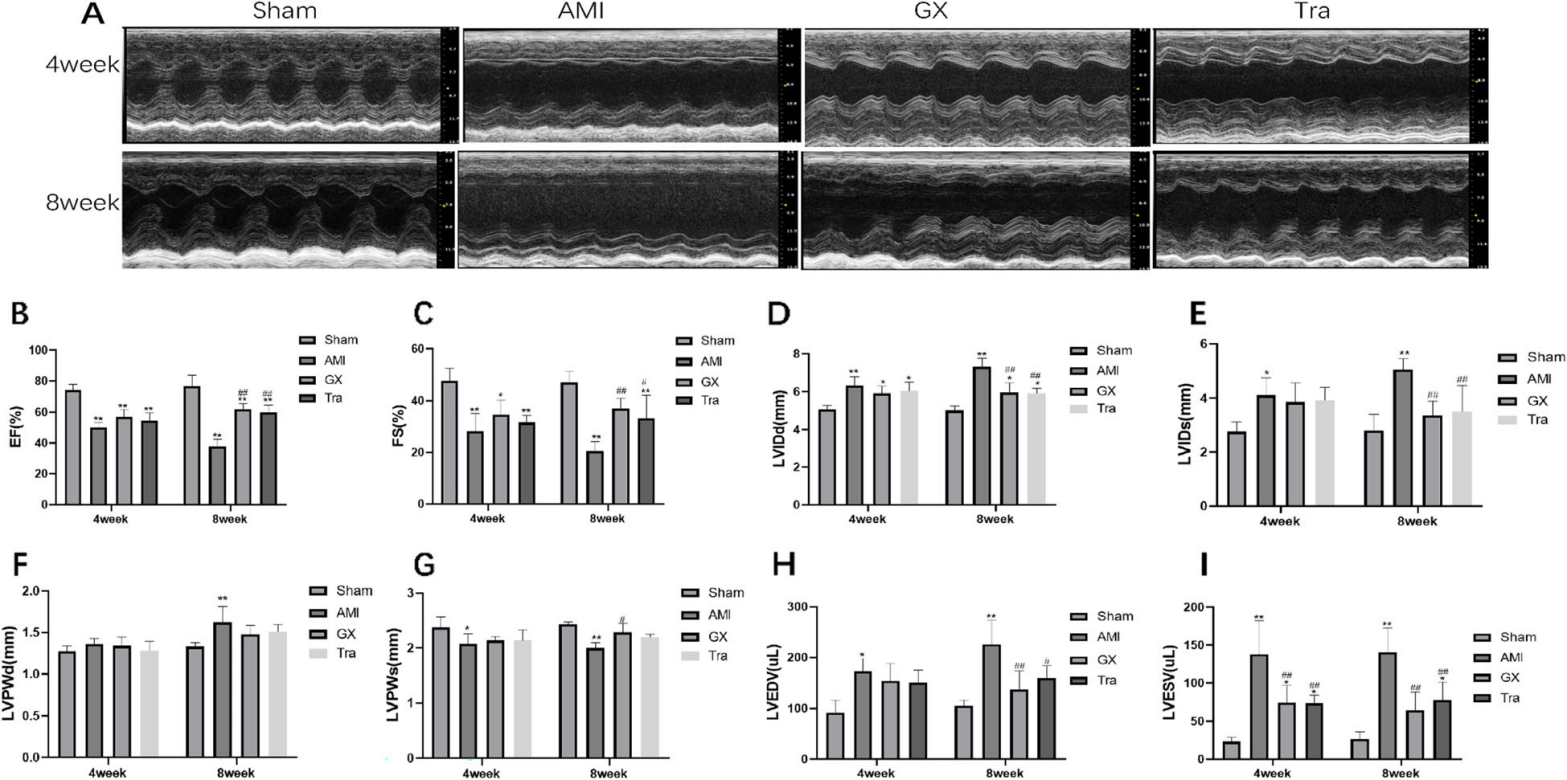
GXV effectively improved cardiac function in AMI hamsters. **a** Representative M-mode echocardiographic images of hamsters in Sham operation, AMI, GXV and Tra groups recorded in the 4th and 8th week, postoperatively (*n* = 6). **b**-**i** EF, FS, LVIDd, LVIDs, LVPWd, LVPWs, LVEDV and LVESV recorded in the 4th and 8th week, postoperatively (*n* = 6). *P < 0.05 *v.s.* Sham operation group; **P < 0.01 *v.s.* Sham operation group. #*P* < 0.05 *v.s.* AMI group; ##*P* < 0.01 *v.s.* AMI group

EF and FS were significantly enhanced after treatment of GXV and Tranilast for 8 weeks compared with those of AMI group (EF: 61.94 ± 3.76% in GXV group and 59.98 ± 4.683% in Tra group *v.s.* 37.82 ± 4.73% in AMI group; FS: 37.07 ± 3.90% in GXV group and 33.03 ± 9.08% in Tra group *v.s.*20.50 ± 3.68% in AMI group). LVIDd and LVIDs were obviously reduced after treatment with GXV and Tranilast in the 8th week (LVIDd: 5.96 ± 0.51 mm in GXV group and 5.92 ± 0.24 mm in Tra group *v.s.* 7.31 ± 0.46 mm in AMI group; LVIDs: 3.37 ± 0.52 mm in GXV group and 3.51 ± 0.94 mm in Tra group *v.s.* 5.03 ± 0.43 mm in AMI group). LVEDV and LVESV were also obviously reduced after treatment with GXV and Tranilast in the 8th week (LVEDV: 137.32 ± 36.01 μL in GXV group and 159.36 ± 24.51 μL in Tra group *v.s.* 226.09 ± 47.31 μL in AMI group; LVESV: 64.29 ± 24.07 μL in GXV group and 77.71 ± 23.67 μL in Tra group *v.s.* 140.83 ± 31.86 μL in AMI group). No significant differences in EF, FS, LVIDd and LVIDs were found between GXV group and Tra group (Fig. 1a-i). Thus, cardiac structure and function were improved after treatment of GXV and Tranilast.

### GXV reduced the myocardial infarction area and HWI

**Fig. 2:**
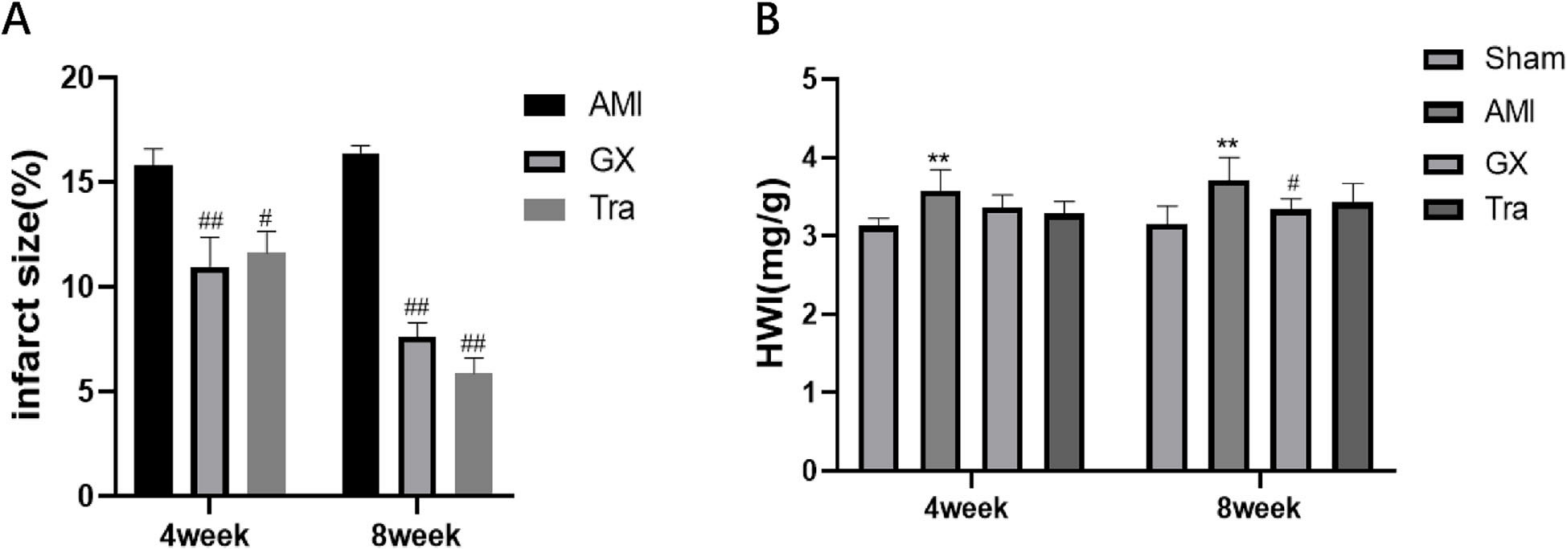
Representative images of TTC staining and analysis of HWI. **a** The infarct size in AMI, GXV and Tra groups in the 4th and 8th week, postoperatively (*n* = 3). **b** HWI in AMI, GXV and Tra groups in the 4th and 8th week, postoperatively (*n* = 6). *P < 0.05 *v.s.* Sham operation group; **P < 0.01 *v.s.* Sham operation group. #*P* < 0.05 *v.s.* AMI group; ##*P* < 0.01 *v.s.* AMI group

The infarcted myocardial tissue was stained to be pale, while the normal part was dark red by TTC. Infarcted size was calculated by the ratio of the infarcted area (pale) and the total area measured by Image J. Notably, infarcted size was obviously narrowed regardless of the treatment for 4 or 8 weeks (*P* < 0.05 and *P* < 0.01, respectively, Fig. 2a). The HWI was calculated as the ratio of the heart weight of body weight. HWI (mg/g) was significantly different between the GXV group and AMI group (3.37 ± 0.15 *v.s.* 3.58 ± 0.27, Fig. 2b). The myocardial infarct size and HWI decreased after treatment of GXV.

### GXV ameliorated the pathological changes and fibrosis in myocardium at post-AMI

**Fig. 3:**
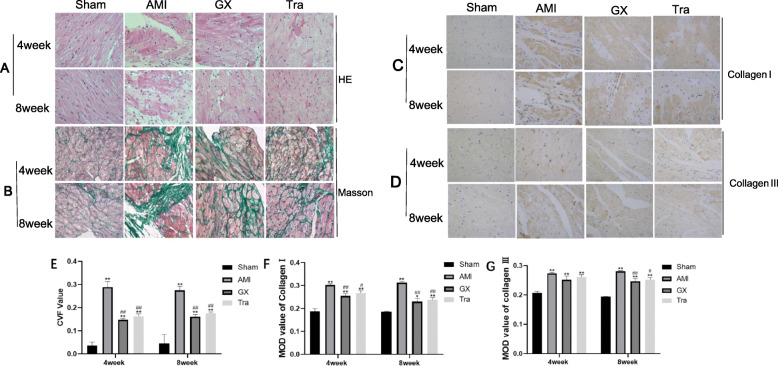
Representative images of H&E, Masson and immunohistochemical staining in myocardium (magnification 400×). **a** H&E staining of myocardium collected from hamsters in Sham operation, AMI, GXV and Tra groups in the 4th and 8th week, postoperatively (*n* = 3). **b**,**e** Masson staining of myocardium collected from hamsters in Sham operation, AMI, GXV and Tra groups in the 4th and 8th week, postoperatively (*n* = 6). Collagen fibers were stained green and myocardial fibers were red. (**c**-**g**) Immunohistochemical staining about positive expressions of Collagen I and Collagen III in myocardium collected from hamsters in Sham operation, AMI, GXV and Tra groups in the 4th and 8th week, postoperatively (*n* = 3). Collagen fibers were stained brown

To observe the function of GXV in alleviating myocardial fibrosis, H&E, Masson and immunohistochemical staining in myocardium were performed. As shown in Fig. 3a, cardiomyocytes in hamsters of Sham operation group were oval. Nuclei were round-shaped and large, which were distributed in the center of cells. Cardiomyocytes were evenly stained and enriched in sarcoplasma. In AMI group, cardiomyocytes were disorderly arranged with significant damages. Nuclei were elongated and interstitial fibrosis was severe. Large-scale apoptosis and necrosis, as well as infiltration of inflammatory cells could be seen. In GXV and Tra group, disorderly arranged cardiomyocytes, interstitial fibrosis and inflammatory response were alleviated (Fig. 3b-g). Cardiac edema was observed. As shown in Fig. 3b, collagenous fiber was dyed green. Collagen I and III were dyed brown in Fig. 3c-d. The CVF value of collagenous fiber, and MOD value of collagen I and III decreased obviously with the treatment of GXV compared with AMI group after 8 weeks (CVF: 0.161 ± 0.011 *v.s.* 0.275 ± 0.017; MOD of collagen I: 0.229 ± 0.014 *v.s.* 0.313 ± 0.005; MOD of collagen III: 0.247 ± 0.008 *v.s.* 0.281 ± 0.002, Fig. 3e-g). The above results all demonstrated that GXV alleviated myocardial fibrosis at post-AMI.

### GXV inactivated the RAAS

**Fig. 4:**
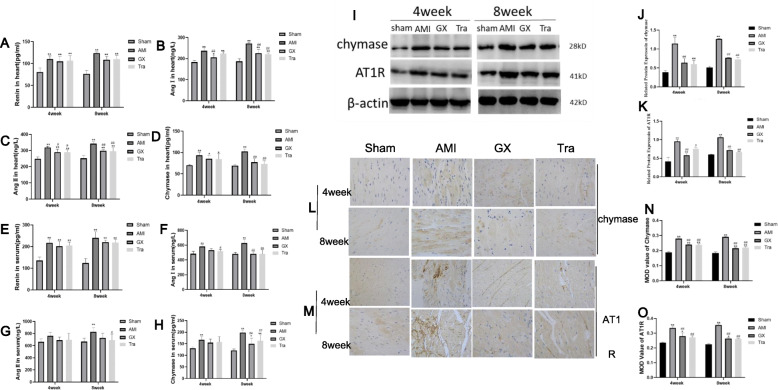
Relative levels of Renin, Ang I, Ang II, chymase and AT1R detected by ELISA, WB and IHC. **a**-**d** Relative levels of Renin, Ang I, Ang II and chymase in heart tissues collected from hamsters in Sham operation, AMI, GXV and Tra groups in the 4th and 8th week, postoperatively (*n* = 5). **e**-**h** Relative levels of Renin, Ang I, Ang II and chymase in serum collected from hamsters in Sham operation, AMI, GXV and Tra groups in the 4th and 8th week, postoperatively (*n* = 6). (**i**-**k**) Protein levels of chymase and AT1R in heart tissues collected from hamsters in Sham operation, AMI, GXV and Tra groups in the 4th and 8th week, postoperatively (*n* = 3). (**l**-**o**) Immunohistochemical staining on chymase and AT1R in heart tissues collected from hamsters in Sham operation, AMI, GXV and Tra groups in the 4th and 8th week, postoperatively (*n* = 3). Magnification: 400 × .*P < 0.05 *v.s.* Sham operation group; **P < 0.01 *v.s.* Sham operation group. #*P* < 0.05 *v.s.* AMI group; ##*P* < 0.01 *v.s.* AMI group

To explore how GXV alleviated myocardial fibrosis after AMI, potential change of the RAAS was detected (Fig. 4). As shown in Fig. 4a and Fig. 4e, the level of Renin had no difference before and after GXV treatment. In particular, relative levels of Ang I, Ang II, AT1R were significantly reduced after treatment. The level of chymase also decreased obviously with the treatment of GXV and Tra. Therefore, in the RASS, the Renin was activated after AMI, but the activation of its products (Ang I, Ang II and AT1R) and chymase was significantly reversed by GXV. It is suggested that GXV protected myocardial fibrosis after AMI by inactivating the RAAS.

### GXV significantly relieved AMI-induced myocardial apoptosis

**Fig. 5:**
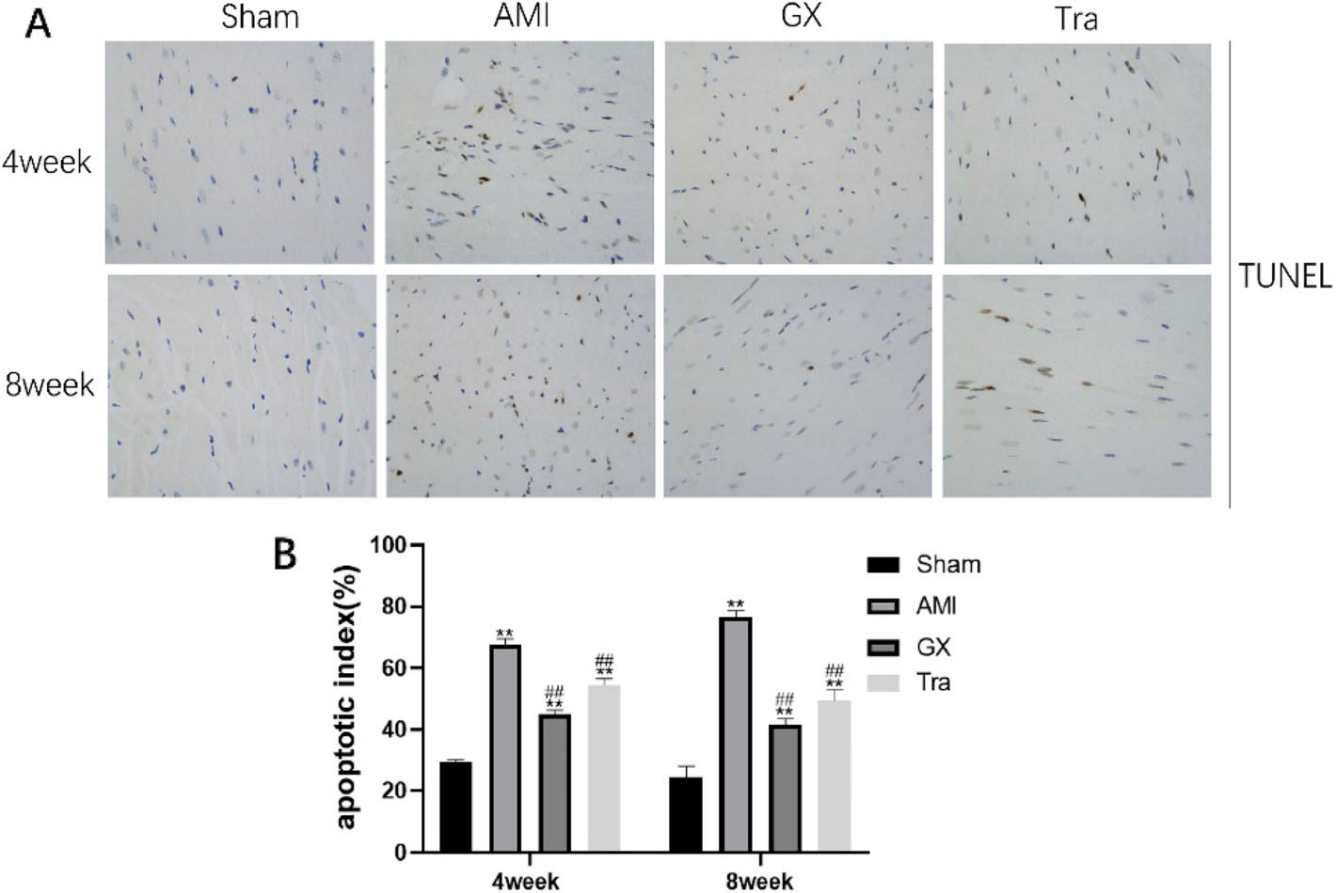
Myocardium apoptosis evaluated by TUNEL assay (magnification 400×). (**a**, **b**) Representative images of TUNEL staining in Sham operation, AMI, GXV and Tra groups in the 4th and 8th week, postoperatively (*n* = 6). Apoptosis cells were stained brown

As shown in Fig. 5, there were a large number of brown-stained apoptosis cells gathered together in the heart tissues from A MI hamsters, whilst the apoptosis was significantly relieved by the treatment of GXV and Tranilast.

### GXV could reduce the number of MCs

**Fig. 6:**
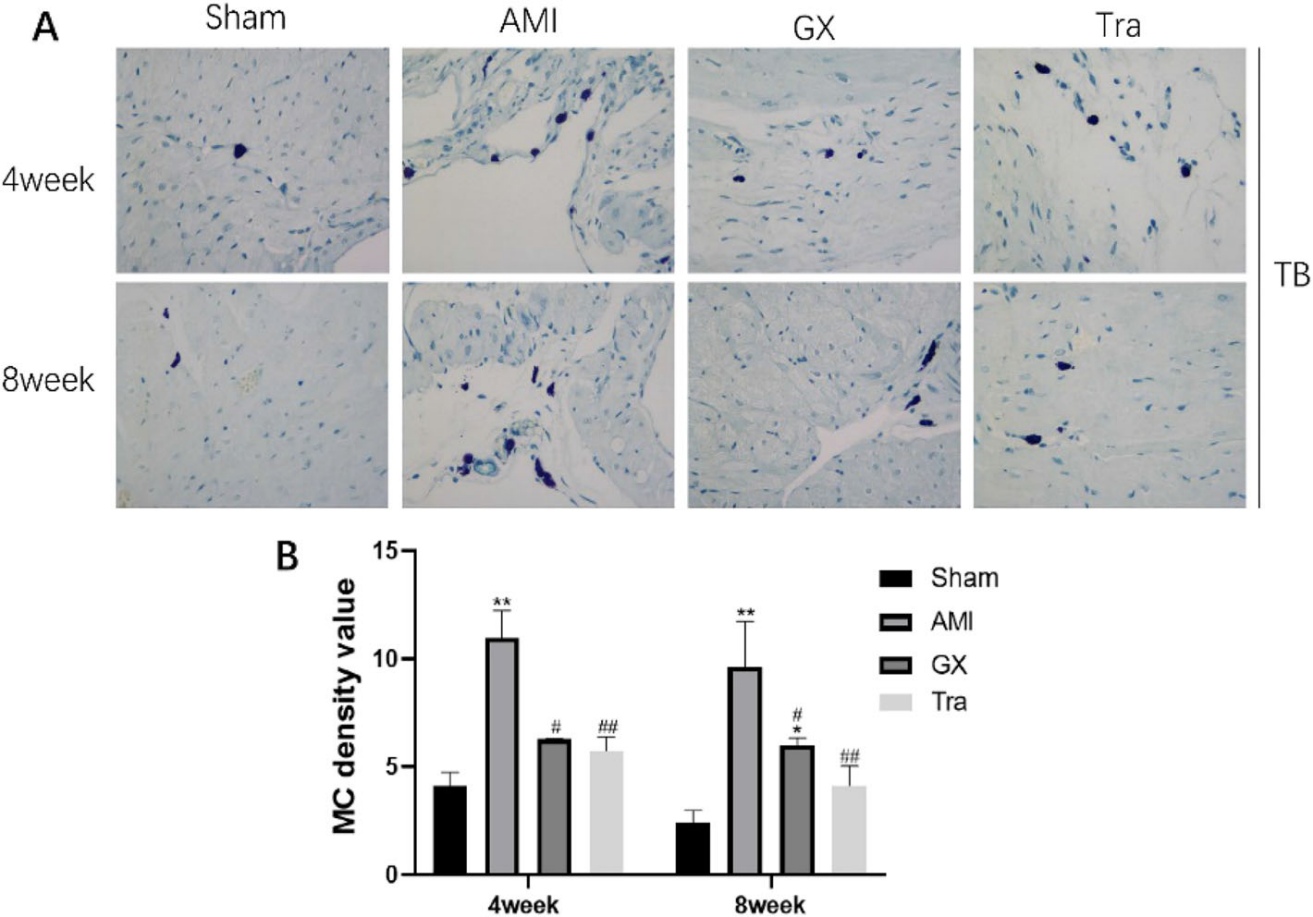
The number of MCs detected by TB staining (magnification 400×). **a**, **b** Representative images of TB staining in Sham operation, AMI, GXV and Tra groups in the 4th and 8th week, postoperatively (*n* = 6). MCs were stained bluish violet

After AMI, the number of MCs was apparently enhanced. Treatment of GXV and Tranilast markedly decreased the number of MCs in hamsters at post-AMI (Fig. 6).

## Discussion

Myocardial infarction and heart failure are major causes of death in the world. A relevant report estimated that the number of patients with heart failure will rise to more than eight million by 2030 [26]. In recent years, the incidence of heart failure at post-AMI has gradually increased. VR plays an important role in the development of myocardial infarction [4]. In the early stage of AMI (several days following AMI), abundant inflammatory cells destroy the collagen scaffold, leading to the expansion of the infarcted myocardium. During this process, fibroblasts are accumulated in the lesioned site, leading to the formation of a new collagen matrix. As a result, the newly formed scar tissues help maintain the structure and shape of ventricles [27–29]. After a few weeks to months, increased load on the surviving cardiomyocytes leads to cardiomyocyte hypertrophy, which eventually results in left ventricular dilatation and progressive changes of mitral valve [30–33]. Once the left ventricle fails to compensate, the left ventricular ejection fraction drops. Therefore, left ventricular diameter and volume can be used as echocardiographic indexes for VR. In this experiment, LVIDd, LVIDs, LVEDV and LVESV were obviously reduced after treatment with GXV and Tranilast in the 8th week.

The activation of RAAS was excessive in the occurrence and development of VR after AMI and can lead to the perpetual vasoconstriction, LV hypertrophy, sympathetic nervous activation [34]. Ang II can either have a direct toxic effect on cardiomyocytes or bind to AT1R. Subsequently, apoptosis, cardiac hypertrophy and production of type I collagen are triggered [35]. The binding between Ang II and AT2R exerts a protective effect on the heart. Their transfection efficacies have been confirmed in some trials, such as the SAVE trial and the OPTIMMAL clinical trial [36–38]. At present, angiotensin-converting enzyme inhibitor (ACEI), angiotensin receptor blockers (ARBs) are commonly used in clinical application to prevent myocardial remodeling following myocardial infarction [39]. However, there are some limitation about these drugs in clinical. ACEI and ARBs can increase the activity of plasma Renin, which are only capable of temporarily reducing plasma aldosterone levels. They cannot block mineralocorticoid receptors. DREAM (Diabetes Reduction Assessment with Ramipril and Rosiglitazone Medication Trial) found that the inhibitory effect of a single treatment with ACEIs on the RAAS is far more less. About 50% of patients with chronic heart failure will develop ACE escape [40]. Therefore, in addition to the classic RAAS, the non-ACE-dependent Ang II production pathway has gradually been concerned. It contains a variety of proteases (e.g. chymase, cathepsin G, Tonin, kallikrein), of which the chymase pathway is the most important.

MCs are abundant in the heart, and they are vital in many diseases, including cancer, allergenic and inflammatory diseases. Specific proteases (including tryptase, chymase, and carboxypeptidase A3) in MCs are encapsulated in granules, which are released through the ligand-dependent pathway during the process of inflammatory response [41]. Growing evidences have indicated that chymase in MCs is one of the pivotal factors leading to tissue fibrosis and remodeling processes via the products of vasoactive and pro-inflammatory substances [42]. Chymases, members of the serine protease family, have a wide range of peptide hydrolysis activities. They are mainly expressed in MCs, fibroblasts, vascular endothelial cells and granulocytes. When AMI occurs, the RAAS is activated and the catalytic activity of chymase is 20 times higher than that of ACE during the transformation from Ang I to Ang II [43]. Chymase distributed in MCs is non-functional, which exerts protease activity only after MCs degranulation under the stimulus of inflammatory cytokines or tissue damages [44]. Numerous studies have supported the role of MCs and chymase in VR and heart failure. Therefore, it is particularly important to develop drugs that can inhibit the activity of chymase and decrease the number of MCs, thereby preventing VR at post-AMI.

In this experiment, echocardiograph findings indicated EF decline and enlargement in the left ventricular cavity in AMI hamsters. Pathological staining results revealed disorderly arranged and damaged cardiomyocytes, large-scale apoptosis and necrosis, inflammatory cell infiltration and severe interstitial fibrosis in the heart of AMI hamsters. Immediately following the onset of AMI, enhanced activity of RAAS, stimulated transformation from Ang I to Ang II and the binding of Ang II to the AT1 receptor induced the release of collagens, further aggravating cardiac fibrosis and VR. During the process, chymase and MCs exerted indispensable roles. After cardiac necrosis, the inflammatory reaction promotes the MCs to release chymase, which catalyzes the transformation of Ang I to Ang II. Notably, GXV treatment for 4 or 8 weeks significantly protected cardiac function and inactivated the RAAS, manifesting as decreased Ang II and collagen levels, alleviated cardiac fibrosis, reduced LV diameter (LVIDd, LVIDs) and volume (LVEDV, LVESV), increased EF and FS. MCs number and chymase level were significantly reduced after treatment with GXV. Similar results were obtained in Tra group as well. It is well known that Tranilast is a specific inhibitor of MCs, and it is effective in delaying ventricular remodeling. Therefore, it was selected as a positive control drug in our experiment. Our findings demonstrated solid evidences that Guanxin V could significantly reduce infarct size, protect cardiac function, reduce cardiac fibrosis and VR at post-AMI, which provided a novel direction in traditional Chinese medicine treatment of AMI. In addition, GXV could reduce the degree of myocardial apoptosis, which protected the cardiac function to a certain extent.

Our study clarified that GXV intervened the RAAS through the non-ACE pathway following AMI. Specific signaling pathways and targets during this process remain largely unclear, which will be further explored in our future experiments.

## Conclusion

GXV protects VR at post-AMI by decreasing chymase level, MCs number and inactivating the RAAS.

## Supplementary Information

**Additional file 1: Table S1.** Summarizes of medicated sera of Guanxin V.

## Data Availability

The datasets generated and/or analyzed during the current study are not publicly available since they are still under further study, but are available from the corresponding author on reasonable request.

## Abbreviations

Ang II: Angiotensin II
Ang I: Angiotensin I
ARBs: Angiotensin receptor blockers
AMI: Acute myocardial infarction
LAD: The ligation of the left anterior descending coronary artery
VR: Ventricular remodeling
GXV: Guanxin V
Tra: Tranilast
LVIDd: Left ventricular end-diastolic inner diameter
LVIDs: Left ventricular end-systolic inner diameter
EF: Ejection fraction
FS: Fractional shortening
LVPWd: Left ventricular posterior wall end-diastolic thickness
LVPWs: Left ventricular posterior wall end-systolic thickness
LVEDV: Left ventricular end diastolic volume
LVESV: Left ventricular end systolic volume
HWI: Heart weight index
TB: Toluidine blue
H&E: Hematoxylin and eosin
WB: Western blotting
PVDF: Polyvinylidene fluoride membranes
ELISA: Enzyme-linked immunosorbent assay
UPLC: Ultra-performance liquid chromatography
AT1R: Angiotensin II type1 receptor
MRAs: Mineralocorticoid receptor antagonists
SNS: Sympathetic nervous system
ECM: Extracellular matrix
MCs: Mast cells
RAAS: Renin-angiotensin-aldosterone System
ACE: Angiotensin converting enzyme
ACEI: Angiotensin-converting enzyme inhibitor
AT2R: Angiotensin II type 2 receptor
TIC: Total ions chromatograph
