# Supplementary material for: Effect of Guanxin V in animal model of acute myocardial infarction
Source: BMC Complement Med Ther. 2021 Feb 22;21:72. doi: 10.1186/s12906-021-03211-7 (PMC7898759; doi:10.1186/s12906-021-03211-7)
Supplement: Supplementary file 1 — Additional file 1: Table S1. Summarizes of medicated sera of Guanxin V. [file 12906_2021_3211_MOESM1_ESM.docx]

**Table S1:** Summarizes of medicated sera of Guanxin V.

| No | RT (min) | Ion mode | Calcd m/z | Observed m/z | Error(ppm) | Formula | MS/MS | Identification status |
| --- | --- | --- | --- | --- | --- | --- | --- | --- |
| 1 | 4.26 | [M+H]^+^ | 416.1921 | 416.1898 | -5.52 | C_19_H_29_NO_9_ | 161.0950[M+H-  C_6_H_10_O_5_-H_2_O-C_3_H_9_NO]^＋^ | Codonopiloside A |
| 2 | 12.59 | [M-H]^-^ | 395.1917 | 395.1942 | 6.33 | C_17_H_32_O_10_ | 263.1505 [M-H-C_5_H_8_O_4_]^−^ | Pentaglucuronol n-hexanol |
| 3 | 12.53 | [M-H]^-^ | 395.1706 | 395.1768 | 15.69 | C_20_H_28_O_8_ | 233.1159 [M-H-C6H10O5]−,  215.1216 [M-HC6H10O5-H2O]− | Lobetyolin |
| 4 | 13.07 | [M+Na]^+^ | 387.1267 | 387.1296 | 7.49 | C_15_H_24_O_10_ | 203. 0870[M+H-Glc]^＋^  173. 1113 [M+H-Glc-CH_2_O]^＋^ | dihydrocatalpol |
| 5 | 3.95 | [M+H]^+^ | 363.1242 | 363.1292 | 13.77 | C_15_H_22_O_10_ | 201.0881 [M+H-Glc]^＋^  171.0662[M+H-Glc-CH_2_O］^＋^ | Ctlpol |
| 6 | 5.24 | [M+H]^+^ | 525.1819 | 525.1854 | 6.66 | C_21_H_32_O_15_ | 325.1028[M+H-HHC]^+^  201.0695 [M+H-DDP]^+^ | RehmanniosideA |
| 7 | 6.42 | [M+CH_3_COO]^+^ | 845.2715 | 845.2720 | 0.59 | C_35_H_46_O_20_ | 625.2473[M+H-Caffeoyl］^+^  163.0073[M+H-DPT］^+^ | Pyroglycoside C / Echinacoside |
| 8 | 6.23 | [M+H]^+^ | 801.2817 | 801.2838 | 2.62 | C_36_H_48_O_20_ | 771.4667[M+H-CH2O]^+^  625.1817[M+H-feruloyl]^+^ | Pyroglycoside A1 /A2 |
| 9 | 5.62 | [M+H]^+^ | 653.2445 | 653.2453 | 1.22 | C_31_H_40_O_15_ | 477.1882[M+H-Feruloyl]^+^  195.0531[M+H-HMP]^+^  177. 0577[M+H-HMP-H_2_O]^+^ | Rehmanniozzzside |
| 10 | 5.62 | [M+H]^+^ | 525.1972 | 525.1954 | -3.43 | C_25_H_32_O_12_ | 195.0531[M+H-HMP]^+^  177. 0577[M+H-HMP-H_2_O]^+^ | 6-O-E-feroyl-ajugalol |
| 11 | 5.16 | [M-H]^-^ | 345.1186 | 345.1203 | 4.93 | C_15_H_22_O_9_ | 183. 0823[M-H-Glc]^-^  165.0251[M-H-Glc-H_2_O]^-^ | Aucubin |
| 12 | 6.35 | [M+Na]^+^ | 399.142 | 399.1473 | 13.27 | C_20_H_24_O_7_ | / | 7R/7S, 8S-guaiacylglycerol-8-O-4'- (coniferyl alcohol) |
| 13 | 4.40 | [M+Cl]^+^ | 407.1109 | 407.1084 | -6.84 | C_17_H_24_O_9_ | / | Syringoside |
| 14 | 4.93 | [M+Na]^+^ | 377.0849 | 377.0828 | -5.57 | C_16_H_18_O_9_ | / | Chlorogenic Acid |
| 15 | 14.65 | [M+H]^+^ | 291.1447 | 291.1429 | -6.18 | C_14_H_22_NO_4_ | / | codonopyrrolidium B |
